# Seroprevalence of Anti-SARS-CoV-2 Remained Extremely Low in Taiwan Until the Vaccination Program Was Implemented

**DOI:** 10.1093/ofid/ofad614

**Published:** 2023-12-14

**Authors:** Yun-Yuan Chen, Min-Hui Yang, Jou-Zhen Lai, Jen-Wei Chen, Yun-Long Wang, Sheng-Tang Wei, Sheng-Mou Hou, Chien-Jen Chen, Ho-Sheng Wu

**Affiliations:** Taiwan Blood Services Foundation, Taipei, Taiwan; Hsinchu Blood Center, Hsinchu, Taiwan; Hsinchu Blood Center, Hsinchu, Taiwan; Taiwan Blood Services Foundation, Taipei, Taiwan; Hsinchu Blood Center, Hsinchu, Taiwan; Taiwan Blood Services Foundation, Taipei, Taiwan; Taiwan Blood Services Foundation, Taipei, Taiwan; Shin Kong Wu Ho-Su Memorial Hospital, Taipei, Taiwan; Academia Sinica, Taipei, Taiwan; Hsinchu Blood Center, Hsinchu, Taiwan; Taipei Medical University, Taipei, Taiwan

**Keywords:** nosocomial infection, SARS-CoV-2, seroprevalence, transfusion safety

## Abstract

**Background:**

The Taiwanese government made a concerted effort to contain a coronavirus disease 2019 (COVID-19) nosocomial outbreak of variant B.1.429, shortly before universal vaccination program implementation. This study aimed to investigate seroprevalence in the highest-risk regions.

**Methods:**

Between January and February 2021, we retrieved 10 000 repository serum samples from blood donors to examine for antibodies against severe acute respiratory syndrome coronavirus 2 (SARS-CoV-2) nucleocapsid (N) and spike (S) antigens. A positive result was confirmed if anti-N and anti-S antibodies were positive. Overall, 2000 donors residing in the highest-risk district and donating blood in January 2021 were further examined for SARS-CoV-2 RNA. We estimated seroprevalence and compared the epidemic curve between confirmed COVID-19 cases and blood donors with positive antibodies or viral RNA.

**Results:**

Twenty-one cases with COVID-19 were confirmed in the nosocomial cluster, with an incidence of 1.27/100 000 in the COVID-affected districts. Among 4888 close contacts of the nosocomial cases, 20 (0.4%) became confirmed cases during isolation. Anti-SARS-CoV-2 was detected in 2 of the 10000 blood donors, showing a seroprevalence of 2/10000 (95% CI, 0.55–7.29). None of the 2000 donors who underwent tests for SARS-CoV-2 RNA were positive. The SARS-CoV-2 infection epidemic curve was observed sporadically in blood donors compared with the nosocomial cluster.

**Conclusions:**

In early 2021, an extremely low anti-SARS-CoV-2 seroprevalence among blood donors was observed. Epidemic control measures through precise close contact tracing, testing, and isolation effectively contained SARS-CoV-2 transmission before universal vaccination program implementation.

Severe acute respiratory syndrome coronavirus 2 (SARS-CoV-2) is the causative agent of coronavirus disease 2019 (COVID-19). It rapidly spread worldwide within months of its recognition, with the World Health Organization (WHO) characterizing the outbreak as a pandemic in March 2020 [1]. Three years later, >670 million cases have been identified, including >6.8 million deaths globally. In May 2023, the Director-General of the WHO declared an end to COVID-19 as a public health emergency “with great hope,” but emphasized that this does not imply that the disease is no longer a global threat.

As an island nation, Taiwan was inevitably forced to face isolation when other countries imposed lockdowns in the early stages of the pandemic, risking shortages of medical supplies and further weakening public health protections. Therefore, the government rapidly and proactively responded to the COVID-19 outbreak during the first 2 years of the pandemic. Before January 2021, in Taiwan, ∼800 cases of COVID-19 were reported, 93% of which were imported. Rigorous border controls, an export ban on critical medical devices, strengthening medical networks, and effective risk communication with the public were the containment procedures [2]. When social distancing could not be maintained, surgical masks were mandatory. Additionally, public health officers immediately launched an investigation to trace all possible close contacts once a patient was laboratory-confirmed [3]. All close contacts were quarantined for 14 days following their last exposure to the index case.

Consequently, before April 2021, small-scale clusters of locally acquired COVID-19 cases in Taiwan were noted [2]. A seroprevalence study conducted in mid-2020 evaluated residual blood samples from ∼15000 patients and observed a 0.07% positivity rate, showing a low cumulative infection rate [4]. However, in January 2021, a nosocomial cluster occurred at a regional hospital in Taoyuan City. The hospital received and treated ∼20% of patients with COVID-19 at that time. Twenty-one locally acquired cases occurred during this outbreak, and 4888 close contacts were isolated. The secondary infection rate among these close contacts was 0.4%. The subgroup strain in this cluster was the Epsilon variant, belonging to lineage B.1.429 [5].

The public health authority had made significant efforts to control nosocomial transmission as the vaccination program was not initiated until March 2021. Symptom-based reverse transcription polymerase chain reaction testing strategies were ineffective in identifying asymptomatic infections or mild cases [6]. Consequently, the reported cases of COVID-19 may only be the tip of the iceberg of undetected transmission [7]. To address the pandemic in Taiwan, adopting measures to block community transmission as much as possible until efficient vaccines and antiviral agents were available was critical. Therefore, we conducted a seroepidemiological study targeting asymptomatic blood donors living in Taoyuan City, which had the highest risk of COVID-19 in Taiwan in early 2021, to evaluate whether SARS-CoV-2 antibody seroprevalence remained low during and after this nosocomial cluster and before the vaccination program implementation. From the standpoint of blood services, we also tested SARS-CoV-2 RNA in some of the blood donors to evaluate the risk of transfusion-transmitted infection.

## METHODS

### Study Design

On January 11, 2021, a nosocomial cluster of COVID-19 was identified at a regional hospital in Taoyuan City. During January and February 2021, 21 new locally acquired cases of COVID-19 were detected in Taiwan, all from this cluster [8]. The demographic characteristics of these confirmed cases are available online [9]. According to the “footprints” of the cases, the Taiwan Central Epidemic Command Center (Taiwan CECC) announced 17 locations related to essential daily necessities for the outbreak. The confirmed cases had visited these places; however, visiting these locations was observed as a potential risk to the general population as not all close contacts were identified (Table 1).

**Table 1.** Footprints of Confirmed Coronavirus Disease 2019 Cases Within the Four Study Districts of Taoyuan City

District	No. of Footprints	Footprints (Locations)
Taoyuan	11	5 restaurants, 3 supermarkets or traditional markets, 1 bakery, 1 café, and 1 hardware store
Luzhu	2	1 supermarket and 1 grocery store
Bade	3	2 supermarkets or traditional markets and 1 bakery
Zhongli	1	1 shopping center

**Figure 1.**
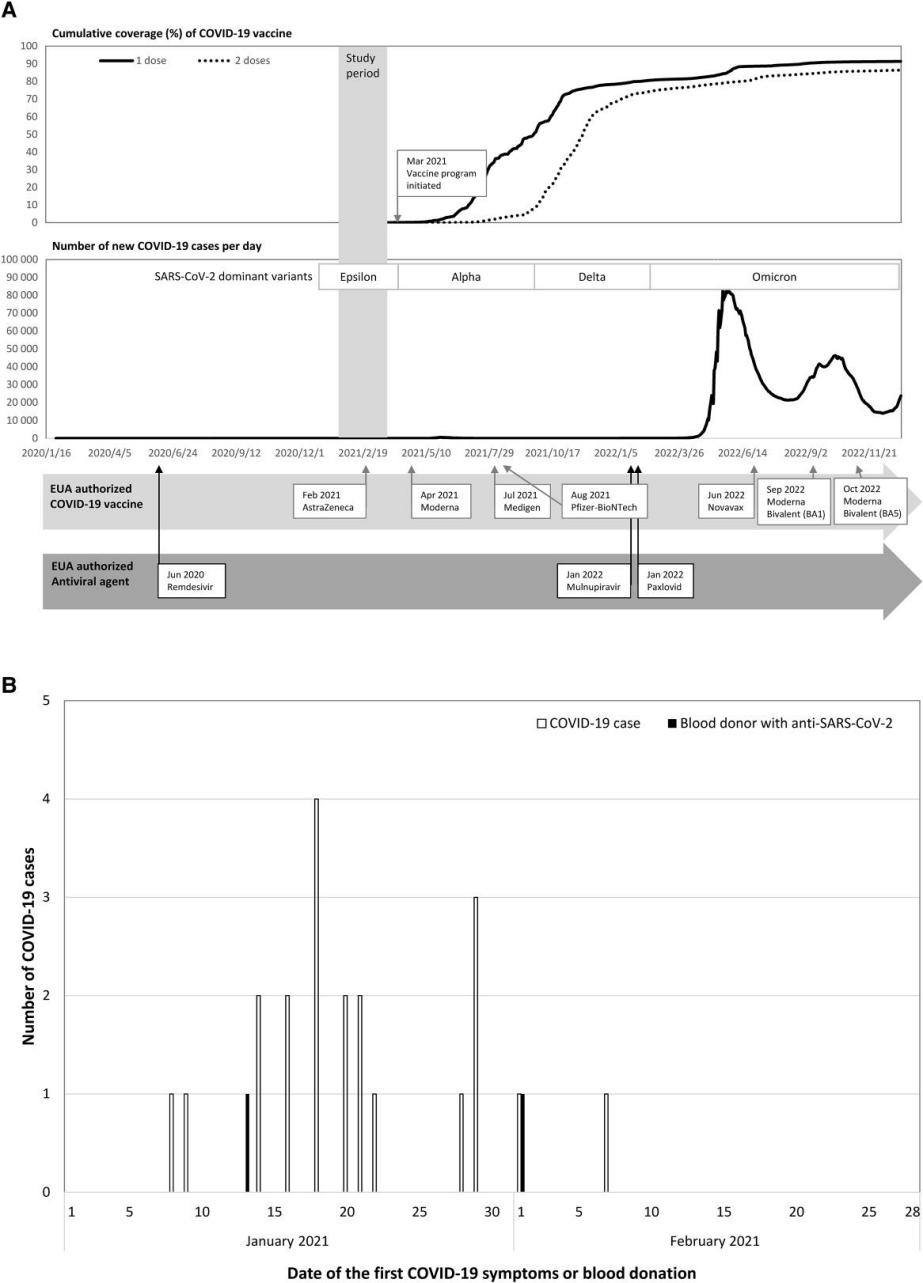
A, Epidemic curve and containment milestones for coronavirus disease 2019 in Taiwan (Numbers were retrieved from OurWorldInData.org) [13]. B, Symptom onset date for 21 confirmed cases [5, 9] and blood donation dates for 2 donors with anti–severe acute respiratory syndrome coronavirus 2 antibodies. Abbreviations: COVID-19, coronavirus disease 2019; EUA, Emergency Use Authorization; SARS-CoV-2, severe acute respiratory syndrome coronavirus 2.

Antibodies can be detected in approximately all COVID-19 cases 3 weeks following infection [10]. Therefore, we conducted a seroprevalence study, selecting blood donations concurrently with the nosocomial outbreak in January to obtain baseline seropositivity and selecting blood donations 1 month following the outbreak in February to reflect the infection rate during the outbreak. Shortly after this outbreak, the COVID-19 vaccination program started on March 22, 2021 (Figure 1A). A total of 10000 blood donors who lived in the 4 affected districts with publicly disclosed footprints of COVID-19 cases and successfully donated blood in Taoyuan City between January and February of 2021 were randomly selected for SARS-CoV-2 antibody testing. These donors represented 87.3% of all blood donors in the affected districts and were randomly selected from each district. The sampling fractions of eligible blood donors living in the Taoyuan, Luzhu, Bade, and Zhongli Districts were 86.3%, 100%, 100%, and 75.5%, respectively, ensuring that each district had a sufficient sample size to obtain accurate rates. Additionally, most patients in this nosocomial outbreak developed symptoms in January, with Taoyuan District having the highest risk due to nosocomial outbreak. Thus, to evaluate the risk of transfusion-transmitted SARS-CoV-2 infection, 2000 of the 10000 blood donors who lived in Taoyuan District and donated in Taoyuan City in January received additional SARS-CoV-2 RNA detection (Figure 1A).

### Study Participants and Serum Specimens

The Taiwan Blood Services Foundation (TBSF) is responsible for blood collection and supply throughout Taiwan [11]. Individuals aged 17 years or older were eligible for blood donation. Beginning in March 2020, blood donors traveling to Wuhan, China, were temporarily deferred for 28 days; this deferral policy was later extended to all donors entering Taiwan from abroad. Furthermore, donors affected by COVID-19 or in close contact with confirmed cases were deferred for 28 days. Additionally, donors with an elevated body temperature (>37.5°C) or those suffering from acute infections were temporarily deferred. During the study period, masks were required to be worn at all times during blood donation, and social distancing and crowd control measures were implemented. Moreover, each blood donation site implemented a system to verify whether a donor had a history of recent overseas travel or close contact with a confirmed COVID-19 case within 30 days using their National Health Insurance card [12].

To identify anti-SARS-CoV-2 and SARS-CoV-2 RNA, serum samples from eligible blood donors in the repository were collected. Since 2009, repository specimens have been collected from each blood donor during blood donation and stored at −20°C for subsequent retrospective investigation. Donor information, including demographic characteristics, details of blood donation, and deferral history after 2020, was obtained from the Database for Evaluating Voluntary Taiwanese Eligible Donors (DEVOTED) operated by the TBSF [11]. All specimens were disconnected in this study.

### Testing for COVID-19

The participants were tested for anti-SARS-CoV-2 using the Elecsys Anti-SARS-CoV-2 Assay (Roche Molecular System Inc., Pleasanton, CA, USA). By using a recombinant protein that represents the nucleocapsid (N) antigen, the assay employs electrochemiluminescence immunoassay and detects pan-immunoglobulin (Ig) antibodies against SARS-CoV-2, including IgM and IgG. The assay has a sensitivity and specificity of 97.2% and 99.8%, respectively [14]. Specimens with a cutoff index of ≥1 were defined as reactive and were further subjected to testing using the Elecsys Anti-SARS-CoV-2 S Assay, which uses a recombinant protein representing the receptor binding domain of the spike (S) antigen. The sensitivity and specificity of the anti-SARS-CoV-2 S assay were 98.8% and 99.98%, respectively [15]. The anti-S assay is a quantitative test, and a positive result is ≥0.8 units/mL. Both measurements were performed using the Roche Cobas E411 automated system (Roch Diagnostics GMBH, Mannheim, Germany). Specimens reactive to both assays were considered anti-SARS-CoV-2-positive, with an overall sensitivity and specificity of 96% and 99.97%, respectively.

SARS-CoV-2 viral RNA was detected using real-time reverse transcriptase polymerase chain reaction (rRT-PCR) using the 1-step LightCycler Multiplex RNA Virus Master Kit assay (Roche Diagnostics, Rotkreuz, Switzerland). In this study, the sequences of the primers and probes used were based on those reported by Croman et al. [16]. The positive controls for the envelope (E) gene, N gene, and RNA-dependent RNA polymerase (RdRp) gene, as well as the Light Mix Modular SARS-CoV (COVID-19) E gene, N gene, and RdRp gene kits, were obtained from Roche Diagnostics. A 70-bp-long fragment from the equine arteritis virus genome was used as a virus extraction control. We first performed an rRT-PCR assay targeting the E and RdRp genes. A cycle threshold value of <40 is considered PCR-positive. If both genes are positive, the test is reported as positive. However, if only 1 gene is positive, it is reported as undetermined, and confirmatory testing is performed using the N gene.

### Statistical Analysis

Incidence rates and corresponding 95% CIs for confirmed cases were estimated using the data announced by the Taiwan CECC. The unadjusted seroprevalence was calculated as the number of reactive specimens divided by the total number of tested specimens using a 95% CI (Wilson score interval). All analyses were performed using SAS software, version 9.4 (SAS Institute Inc., Cary, NC, USA).

## RESULTS

At the beginning of 2021, 21 cases of locally acquired COVID-19 in the nosocomial cluster were noted, resulting in an incidence of 1.27 per 100000 individuals in the 6 affected districts within Taoyuan City. Fourteen (66.7%) cases were female, and 12 (57%) were aged ≥50 years. Taoyuan District had the highest number of cases, followed by Luzhu, Bade, Guishan, Zhongli, and Pinzhen Districts, with corresponding incidence rates of 2.41, 2.39, 0.96, 0.61, 0.47, and 0.44 per 100000 individuals, respectively (Table 2). The 4 districts with the highest incidence, Taoyuan, Luzhu, Bade, and Zhongli, accounted for 90.5% of all COVID-19 cases in the nosocomial cluster. Moreover, 9 (47.4%) cases had footprints occurring in locations where they purchased daily necessities in these 4 districts.

**Table 2.** Confirmed Cases and Incidence of COVID-19 in January and February 2021

Characteristics	Six Affected Districts				Four Study Districts			
	No.	%	Incidence (1/10^5^)		No.	%	Incidence (1/10^5^)	
			Rate	95% CI			Rate	95% CI
Total	21	100.0	1.27	0.83–1.95	19	100.0	1.51	0.97–2.36
Sex								
Male	7	33.3	0.86	0.42–1.79	7	36.8	1.14	0.55–2.35
Female	14	66.7	1.67	0.99–2.80	12	63.2	1.87	1.07–3.27
Age								
<20 y	0	0.0	0.00	…	0	0.0	0.00	…
20–29 y	4	19.0	1.78	0.69–4.59	4	21.1	2.35	0.91–6.05
30–39 y	3	14.3	1.13	0.38–3.32	2	10.5	1.00	0.27–3.65
40–49 y	2	9.5	0.72	0.20–2.62	2	10.5	0.94	0.26–3.42
50–59 y	5	23.8	2.10	0.90–4.91	4	21.1	2.19	0.85–5.63
60+ y	7	33.3	2.27	1.10–4.69	7	36.8	3.00	1.46–6.20
District								
Taoyuan	11	52.4	2.41	1.34–4.31	11	57.9	2.41	1.34–4.31
Luzhu	4	19.0	2.39	0.93–6.16	4	21.1	2.39	0.93–6.16
Bade	2	9.5	0.96	0.26–3.49	2	10.5	0.96	0.26–3.49
Zhongli	2	9.5	0.47	0.13–1.73	2	10.5	0.47	0.13–1.73
Pingzhen	1	4.8	0.44	0.08–2.48	…	…	…	…
Guishan	1	4.8	0.61	0.11–3.45	…	…	…	…

Abbreviation: COVID-19, coronavirus disease 2019.

Overall, 10000 blood donation specimens were tested for anti-SARS-CoV-2, and 2 of them tested positive, for a positivity rate of 2 per 10000 individuals (95% CI, 0.55–7.29). This rate was much lower than the secondary infection rate of 40.9 per 10000 individuals (95% CI, 26.5–63.1) among 4888 close contacts. Additionally, comparing the rates between January (1.79 per 10000 individuals; 95% CI, 0.32–10.13) and February (2.27 per 10000 individuals; 95% CI, 0.40–12.83) 2021, the seroprevalence did not significantly increase during the study period (*P* = .49). Of the confirmed COVID-19 cases in this nosocomial cluster, the first and last cases had symptom onset on January 8 and February 7, respectively. On January 18, the peak of the epidemic occurred (Figure 1B), and 2 blood donors, who donated blood on January 13 and February 1, respectively, were noted with positive anti-SARS-CoV-2 results. Therefore, compared with this nosocomial cluster, SARS-CoV-2 infection was rarely observed in blood donors.

The characteristics of the 10000 blood donors are shown in Table 3. A total of 87.3% of the blood donors who lived in the 4 districts and donated in Taoyuan City between January and February 2021 were included. Among them, 5688 (56.9%) were male, and 3347 (33.5%) were aged ≥50 years. Approximately 98% (n = 9754) of the selected specimens were obtained from whole-blood donors. The proportion of blood donors living in the Taoyuan District was 38.9% (n = 3892). Only 135 donors were deferred from blood donation owing to colds (0.81%) or traveling abroad (0.55%) between January 2020 and February 2021. The 2 antibody-positive donors were male, whole-blood donors, <50 years old, and living in the Taoyuan District. Neither of them had been deferred because of a cold or traveling abroad after 2020.

**Table 3.** Serum Anti-SARS-CoV-2 Test Results Among 10 000^a^ Blood Donors Living in the Four Districts of Taoyuan City

Characteristics	Total (n = 10 000)		Anti–SARS-CoV-2 (+)		
	No.	%	No.	Prevalence (1/10 000)	
				Rate	95% CI
Age					
<20 y	167	1.67	0	0.00	…
20–29 y	1287	12.87	1	7.77	1.37–43.88
30–39 y	2211	22.11	0	0.00	…
40–49 y	2988	29.88	1	3.35	1.84–24.37
50–59 y	2445	24.45	0	0.00	…
60+ y	902	9.02	0	0.00	…
Sex					
Male	5688	56.88	2	3.52	0.96–12.81
Female	4312	43.12	0	0.00	…
District					
Taoyuan	3892	38.92	2	5.14	1.41–18.72
Luzhu	1934	19.34	0	0.00	…
Bade	1607	16.07	0	0.00	…
Zhongli	2567	25.67	0	0.00	…
Date of donation					
January 1–15	2234	22.34	1	4.48	0.79–25.31
January 16–31	3354	33.54	0	0.00	…
February 1–15	1349	13.49	1	7.41	1.31–41.87
February 16–28	3063	30.63	0	0.00	…
Donation site					
Fixed site	5470	54.70	1	1.83	0.32–10.35
Mobile drives	4530	45.30	1	2.21	0.39–12.49
Type of donation					
Whole blood (1U)	5354	53.54	1	1.87	0.33–10.57
Whole blood (2U)	4400	44.00	1	2.27	0.40–12.86
Platelet apheresis (1U)	88	0.88	0	0.00	…
Platelet apheresis (2U)	158	1.58	0	0.00	…
Deferrals (cold)^b^					
Yes	81	0.81	0	0.00	…
No	9919	99.19	2	2.02	0.18–5.71
Deferrals (travel abroad)^b^					
Yes	55	0.55	0	0.00	…
No	9945	99.45	2	2.01	0.18–5.69

Abbreviation: SARS-CoV-2, severe acute respiratory syndrome coronavirus 2.

^a^Of the 10000 blood donors, 2000 were from the Taoyuan District, and polymerase chain reaction tests were simultaneously performed.

^b^From January 1, 2020, blood donors were subjected to restricted blood donation because of cold or returning from abroad.

To detect SARS-CoV-2 RNA in the 2000 blood donors who lived in the Taoyuan District and donated blood in January 2021, we performed additional PCR tests; all tested negative. Their demographic distribution was similar to that of the other 8000 blood donors (Table 4).

**Table 4.** Serum SARS-CoV-2 RNA and Antibody Testing Results Among 2000 Blood Donors in Taoyuan District, Taoyuan City

Characteristics	Total (n = 2000)		RNA (+)	Antibody (+)
	No.	%	No.	No.
Age				
<20 y	25	1.3	0	0
20–29 y	286	14.3	0	1
30–39 y	426	21.3	0	0
40–49 y	616	30.8	0	0
50–59 y	489	24.5	0	0
60+ y	158	7.9	0	0
Sex				
Male	1190	59.5	0	1
Female	810	40.5	0	0
Date of donation				
January 1–15	831	41.6	0	1
January 16–31	1169	58.5	0	0
Donation site				
Fixed site	1612	80.6	0	0
Mobile drives	388	19.4	0	1
Type of donation				
Whole blood (1U)	996	49.8	0	1
Whole blood (2U)	933	46.7	0	0
Platelet apheresis (1U)	28	1.4	0	0
Platelet apheresis (2U)	43	2.2	0	0
Deferrals (cold)^a^				
Yes	15	0.8	0	0
No	1985	99.3	0	1
Deferrals (travel abroad)^a^				
Yes	15	0.8	0	0
No	1985	99.3	0	1

Abbreviation: SARS-CoV-2, severe acute respiratory syndrome coronavirus 2.

^a^From January 1, 2020, blood donors were subjected to restricted blood donation because of cold or returning from abroad.

## DISCUSSION

Our study showed that unvaccinated blood donors in Taiwan had an extremely low anti-SARS-CoV-2 seroprevalence 1 year following the start of the pandemic, even in areas with the highest COVID-19 risk. This result also demonstrated the low spread of SARS-CoV-2 based on seroepidemiological evidence. It revealed the success of a series of containment measures in this vulnerable island country during the early stages of the pandemic [12]. Serological surveillance is a useful tool for determining the cumulative spread of SARS-CoV-2 infection [17]. However, the true number of SARS-CoV-2 infections is largely underestimated by symptom-based screening in health care settings [6, 18]. The TBSF regularly collects blood specimens from blood donors, which can serve as a source for population-based epidemiological surveillance.

Owing to the multitude of eligibility criteria for blood donation, blood donors may be at a lower risk of infection than the general population; therefore, the seroprevalence in this study may have been underestimated. During the study period, overseas travel was the main source of exposure to SARS-CoV-2, and the nosocomial outbreak in Taoyuan City started from an imported case. Therefore, inbound passengers, patients with COVID-19, and individuals who had close contact with patients with COVID-19 were all required to quarantine or self-isolate (stay at home) for 14 days, and blood donations were temporarily deferred for 28 days, thereby delaying their scheduled blood donations. The quarantine and self-isolation policies protected all vulnerable populations; therefore, blood donors were at the same risk of exposure to SARS-CoV-2 as the general population. Furthermore, the deferral period was long enough for individuals with infection to produce sufficient antibodies for antibody testing; therefore, to reflect the infection during the outbreak and obtain a more accurate rate, we included blood donations performed 1 month following the outbreak. SARS-CoV-2 is primarily transmitted through the respiratory tract. The infection itself is at low risk for transfusion-transmitted infection. Viral RNA was detectable in 10% of the patients with COVID-19 [19, 20], which correlated with disease severity [18, 21]. However, to the best of our knowledge, there have been no reported cases of COVID-19 transmission through blood transfusions. In our study, all of the 2000 blood donors living in the district with the highest COVID-19 incidence tested negative for SARS-CoV-2 RNA, indicating that the risk of transfusion-transmitted infection can be nullified in a low-risk population.

The seroprevalence of SARS-CoV-2 antibodies has rapidly and significantly increased in several countries within 1 year following the WHO declaring COVID-19 a pandemic. From the start of 2020 to mid-2020, the anti-SARS-CoV-2 prevalence rates among blood donors in Canada [22], the Netherlands [23], Kenya [24], and Brazil [25] were 0.7%, 5.9%, 8.0%, and 13.6%, respectively. In the general population during the same period, the seroprevalence rates were 1.31%, 5.0%, 7.8%, and 18.6% in Austria [26], Spain [27], Switzerland [28], and Russia [29], respectively. Notably, between July 2020 and May 2021, the estimated seroprevalence among >1.4 million blood donor samples in the United States increased from 3.5% to 20.2% [30]. Likewise, in December 2020, the seroprevalence rate in the general Russian population sharply increased to 39.8% [29]. Starting in March 2020, all of these countries closed their borders or allowed entry only to their citizens. Most countries implemented lockdowns following the surge in March 2020; these measures have proven effective in controlling the outbreak [26, 31]. However, individuals who had been in close contact with patients with COVID-19 in these countries were not subjected to mandatory quarantine. This may be detrimental to outbreak control, as 1 study noted that 48% of the individuals who tested positive for anti-SARS-CoV-2 antibodies were not suspected of being infected with SARS-CoV-2 [23].

In contrast, in 2020, the seroprevalence rates in Taiwan were 0.4% among patients at epidemiological risk [32], 0.07% among ∼15 000 patients in a tertiary care center [4], and 0.083% among 4841 high-risk individuals in central Taiwan [33]. All of these rates were lower than the secondary clinical attack rate of 0.7% among close contacts [3]. Our study included several healthy blood donors of various ages living in high-risk areas in early 2021 and observed that seroprevalence was extremely low compared with other countries in 2020. One of Taiwan's precision public health measures launched in late 2019 was to implement on-board quarantine on flights returning to Taiwan from Wuhan, China, ≥2 months ahead of other countries' control measures. This approach identified the first confirmed case of imported COVID-19 and successfully contained the virus outside its borders. In January 2020, a 14-day mandatory preventive quarantine program was subsequently implemented for confirmed COVID-19 cases, close contacts of confirmed cases, and inbound travelers from areas at higher risk for COVID-19. Shortly thereafter, to quickly identify and isolate COVID-19 cases and their close contacts, the Taiwan Centers for Disease Control conducted comprehensive contact tracing [2, 3].

As of the end of 2021, a total of 93 721 close contacts, 950 138 inbound travelers, and 1 075 176 individuals were subjected to home isolation, quarantine, and self-management, respectively, and the COVID-19 confirmed rates were 7.77%, 0.13%, and 0.05%, among these populations, respectively. Violations were observed in only 0.12% (2497/2 119 035 individuals). Furthermore, our results reflect the robustness and sensitivity of the Taiwan National Notifiable Diseases Surveillance System [34]. In Taiwan, within 3 months following the start of the pandemic, the median time from COVID-19 onset to notification was reduced from 5 days to 1 day, which is key to the timely detection of cases and isolation of close contacts.

Individuals in Taiwan were able to go to work and school normally during the pandemic, without being blocked or restricted by movement, through these effective stringent control measures. Our study demonstrated that these measures were effective in preventing community transmission of SARS-CoV-2 in the highest-risk areas before the immunization program was implemented (Figure 1A). Our population had built herd immunity to SARS-CoV-2 primarily through vaccination, and we strived for more time to expand medical capacity, COVID-19 treatment experiences, and antiviral stockpiles. These stringent measures were undertaken to protect vulnerable populations in a geographically isolated country from the devastation caused by the 2003 SARS outbreak, which risked shortages of critical medical supplies early in the outbreak. After 3 major waves of Omicron outbreaks and full vaccination coverage of >85% of the population, the Taiwan CECC announced the cancelation of mandatory isolation and reporting of mild COVID-19-confirmed cases starting in March 2023.

This study had some limitations. First, the observed seroprevalence may have been underestimated. During blood donation, blood donors must be asymptomatic. Throughout the study period, if a blood donor had been in close contact with a confirmed case, blood donation had to be deferred for 28 days. Additionally, the prevalence [27] and levels [10, 35] of SARS-CoV-2 antibodies were higher in severely ill patients than in mildly ill or asymptomatic patients. Furthermore, not all individuals infected with SARS-CoV-2 develop an antibody response. It is estimated that 81.1% of asymptomatic patients test positive for IgG antibodies ∼3–4 weeks following exposure [10]. Conversely, as the blood donors were all from Taoyuan City, which had only locally acquired COVID-19 cases from January to February 2021, geographical bias may have existed. This may overestimate the seroprevalence compared with donor populations in other regions.

In conclusion, Taiwan's precision public health measures including prompt implementation of border controls and thorough identification and isolation of COVID-19 cases as quickly as possible successfully limited the spread of SARS-CoV-2 in Taiwan 1 year following the pandemic. The success of these measures allowed more time for the development of vaccines and antiviral agents, making it possible to address novel variants with better public health protection, and helped individuals to smoothly pass through all stages of the pandemic. Therefore, Taiwan's experience in fighting the COVID-19 pandemic can serve as a model for other geographically isolated countries/regions worldwide to rapidly and proactively respond to future emerging infectious diseases.
